# Predicting treatment outcomes of pain patients attending tertiary multidisciplinary pain treatment centers: A pain trajectory approach

**DOI:** 10.1080/24740527.2017.1325715

**Published:** 2017-08-04

**Authors:** M. Gabrielle Pagé, E. Manolo Romero Escobar, Mark A. Ware, Manon Choinière

**Affiliations:** a Centre de Recherche du Centre hospitalier de l’Université de Montréal (CRCHUM), Montreal, Quebec, Canada; b Department of Biomedical Sciences, Faculty of Medicine, Université de Montréal, Montreal, Quebec, Canada; c Department of Psychology, Faculty of Health, York University, Toronto, Ontario, Canada; d Department of Family Medicine, McGill University, Montreal, Quebec, Canada; e Departement of Anesthesia, Faculty of Medicine, McGill, Montreal, Quebec, Canada; f Alan Edwards Centre for Research on Pain, McGill University, Montreal, Quebec, Canada; g Department of Anesthesiology, Faculty of Medicine, Université de Montréal, Montreal, Quebec, Canada

**Keywords:** pain trajectories, chronic pain, multidisciplinary pain treatment facility, Quebec Pain Registry

## Abstract

**Background**: Though multidisciplinary pain treatment (MPT) is considered the gold standard for managing chronic pain, it is unclear which patients benefit most from this high-cost treatment approach.

**Aims**: The goals were to identify subgroups of patients sharing similar pain severity trajectories over time and predictors of MPT responsiveness.

**Methods**: Participants were 1894 patients (mean age = 53.18 years [SD = 14.0]; female = 60.3%) enrolled in the Quebec Pain Registry with moderate to severe baseline pain severity. Patients completed validated questionnaires on pain and related constructs before initiating treatment and 6, 12, and 24 months later.

**Results**: Trajectory analyses of pain severity (intensity and interference) showed that a three-class model best fit the data. Two of the trajectories, which included 24.5% of patients, showed significant improvement in pain severity levels over time (improvers). Compared to patients in the nonimproving trajectory (non-improvers), improvers were younger and more likely to suffer from neuropathic pain and had pain of shorter duration, lower worst pain intensity, lower sleep disturbances and depression scores at baseline, a lower tendency to catastrophize, and better physical health–related quality of life (QOL). This predictive model had a specificity of 96.2% and a sensitivity of 23.6%.

**Conclusions**: Only a minority of patients exhibited an improvement in their pain severity with MPT. Several patients’ characteristics were significantly associated with pain trajectory membership. Early identification of nonimprovers, through examination of baseline characteristics and rates of change in pain scores, can provide valuable information about prognosis and open the doors for evaluation of different cost-effective treatment approaches.

**Abbreviations**: CP = chronic pain; MPT = multidisciplinary pain treatment; QPR = Quebec Pain Registry; QOL = quality of life.

## RéSUMé

**Contexte**: Bien que le traitement multidisciplinaire de la douleur soit considéré le modèle d’excellence de la prise en charge de la douleur chronique, on ne sait pas exactement qui sont les patients qui bénéficient le plus de cette approche thérapeutique relativement coûteuse. **Objectifs**: L’étude visait à identifier des sous-groupes de patients présentant des trajectoires similaires en ce qui concerne la sévérité de leur douleur au fil du temps, de même que les facteurs prédictifs de la réponse au traitement multidisciplinaire de la douleur. **Méthodes**: Un total de 1 894 participants (âge moyen = 53,18 ans (ÉT = 14,0); femmes = 60,3 %), étaient inscrits au Registre Québec douleur et souffraient de douleur modérée à sévère au avant le début du traitement. Les patients ont répondu à des questionnaires validés portant sur la douleur et des concepts connexes avant de commencer le traitement, puis six, douze et vingt-quatre mois plus tard. **Résultats**: Les analyses de trajectoire de la sévérité de la douleur (intensité et interférence) ont démontré qu’un modèle à trois catégories convenait le mieux aux données. Deux des trajectoires, qui comprenaient 24,5 % des patients, ont montré une amélioration importante des niveaux de sévérité de la douleur au fil du temps (patients avec amélioration). Comparativement aux patients de la trajectoire sans amélioration, les patients avec amélioration étaient plus jeunes et plus susceptibles de souffrir de douleur neuropathique, leur douleur était présente depuis moins longtemps et l’intensité de leur pire douleur était moindre. De plus, ils étaient moins susceptibles de souffrir de perturbations du sommeil, leurs scores de dépression étaient inférieurs avant le début du traitement et ils avaient moins tendance à catastrophiser, en plus d’avoir une meilleure qualité de vie liée à leur santé physique. Ce modèle prédictif avait une spécificité de 96,2 % et une sensibilité de 23,6 %. **Conclusion**: Seulement une minorité de patients ont démontré une amélioration de la sévérité de la douleur avec le traitement multidisciplinaire de la douleur. Une association significative a été établie entre les caractéristiques de plusieurs patients et leur appartenance à l’une ou l’autre des trajectoires de la douleur. L’identification précoce des patients sans amélioration par l’examen de leurs caractéristiques avant le début du traitement et des taux de changement dans leurs scores de la douleur peut fournir des renseignements utiles pour le pronostic et ouvrir la porte à l’évaluation de différentes approches de traitement qui présentent un bon rapport coût-efficacité.

## Introduction

The prevalence of chronic pain (CP), namely, pain that persists beyond normal tissue healing time or is associated with a chronic medical disorder (>3–6 months),^1^ is around 20% in the general population.^2–4^ CP also presents an important economic burden, costing between US$560 and US$635 billion yearly in terms of direct and indirect costs.^5^ Though certain patients recover from their pain experience, a significant number of them require multidisciplinary pain treatment (MPT). MPT is considered to be the optimal standard of care for CP^6–10^; however, the waiting time to access such specialized services can be as long as 2 years.^11,12^

A review of published reports on the effectiveness of MPT in specialized clinics^10^ has shown that patients report (1) a reduction in their pain intensity varying between 14% and 60%, with an average reduction of 20%–30%, (2) a significant improvement (65%) in physical activity, and (3) an average return to work rate of 66% following treatment. In addition, decreased health care utilization in the year following attendance at an MPT clinic has been reported; studies have shown that between 60% and 90% of patients do not seek additional health care in the first year following treatment completion whereas costs associated with treating CP decrease by 68% after treatment.^10^

These statistics provide strong evidence as to the effectiveness of MPT for a large proportion of patients, yet little is known about which patients will respond most to treatment. The current literature on MPT is limited by the fact that methodological approaches examining treatment effectiveness typically use average pain scores across patient groups, resulting in the loss of between- and within-individual variations in pain experience.^13^ Research has repeatedly shown methodological issues with using such a measurement approach and recommended that multiple time points be used to increase accuracy and take into account interindividual variability in predicting prognosis.^14–18^

The overall aim of this study was to examine pain severity trajectories over a period of 24 months among patients suffering from moderate to severe pain who attended an MPT clinic. The specific objectives of this study were to (1) examine inter- and intra-individual variations in pain severity (pain intensity and interference) trajectories over a 2-year period; (2) identify baseline pain and psychological characteristics that predicted trajectory membership; and (3) determine whether types of pain trajectories can predict patient outcomes at 24 months.

## Materials and methods

### Participants

Study participants were selected from patients enrolled in the Quebec Pain Registry^19^ (QPR; http://www.quebecpainregistry.com) who provided written consent for their QPR data to be used for research purposes (91.4% of patients). The QPR was developed and implemented to monitor the condition of patients suffering from various types of pain syndromes who were referred to large MPT clinics (dedicated centers of expertise) in the province of Quebec (Canada) using common demographics, identical clinical descriptors, and uniform outcome measures. Patients were enrolled in the QPR if they were (1) scheduled for a first visit at the pain clinic for multidisciplinary treatment considerations, (2) aged 18 years or older, (3) fluent in spoken and written French and/or English, and (4) physically or cognitively able to complete questionnaires. Patients were excluded if they were eligible for recruitment in the preexisting Fibromyalgia Registry at one of the participating sites. Patients seen at an MPT clinic were offered different treatment options based on their clinical profile. Treatment was thus individualized to patient needs. Treatments could include one or a combination of the following treatments: pharmacotherapy, physiotherapy, psychotherapy, chiropractic care, and interventions (e.g., blocks).

Patients suffering from chronic non-cancer pain (≥3 months), reporting baseline pain intensity and/or interference scores in the moderate to severe range (Numeric Rating Scale [NRS-11] ≥ 4 for pain intensity on the average in the past 7 days^20^; Brief Pain Inventory [BPI-10] mean score ≥ 4 on the pain interference scales) were included in the present study. Patients with mild pain intensity and interference were excluded from the analyses. Due to floor effects, these patients could not show an improvement in their pain severity to the same extent as those who started with higher pain levels.

In addition, to be included in the present study, participants had to have been enrolled in the QPR between November 2008 (date of implementation of the QPR) and March 2011. Patients entering the QPR after March 2011 were excluded because follow-up data were no longer collected at 12 and 24 months starting in April 2012 (patients enrolled after March 2011 did not reach the 12-month follow-up before these changes were made and thus did not complete any of the 12- and 24-month follow-ups). Participants attended one of the three designated tertiary clinics of the Quebec Pain Centres of Expertise located at the Centre hospitalier de l’Université de Montréal (CHUM), McGill University Health Centre (MUHC), and Centre hospitalier de l’Université de Sherbrooke (CHUS).

### Procedure

The QPR project was approved by the institutional research ethics boards of the CHUM, MUHC, and CHUS. Successive patients who came for a first appointment at one of the participating pain clinics were enrolled in the QPR. They were informed that the information collected as part of the QPR had both clinical (production of a summary report of their clinical condition for the physician with whom they had an appointment) and administrative (e.g., generation of annual statistical reports) purposes. Patients were invited to sign the research ethics board–approved consent form if they agreed to the use of their QPR data for research purposes.

Biopsychosocial data (e.g., pain intensity and interference, emotional well-being, health-related quality of life) were collected with a self-report questionnaire (patient self-administered questionnaire) and medical/clinical data (e.g., pain duration, pain diagnosis, treatments, etc.) were gathered by the QPR nurses using a structured interview protocol (nurse-administered questionnaire) prior to the patient’s first appointment at the pain clinic (baseline). Participants answered the patient and the nurse questionnaires at baseline and 6 months later. Additional follow-up measures were collected at 12 and 24 months but only in patients who had not been discharged from the pain clinic in the meantime. If a patient was discharged within the first 6 months following her or his first appointment, follow-up ended at 6 months. If this was the case between 6 and 12 months, follow-up was carried out at 12 months but not at 24 months to minimize participant burden.

### Questionnaires and measures

#### Numeric Rating Scale for pain intensity

The NRS^21^ is a self-administered scale that measures pain intensity and ranges from 0 (*no pain at all*) to 10 (*worst possible pain*). The NRS has been shown to have good reliability, validity, and sensitivity to change.^21^ Participants were asked to rate their current pain intensity, average pain intensity in the past 7 days, and worst pain intensity during the same period.

#### Type of pain

Douleur Neuropathique 4 Questions (DN4)^22^ is a screening diagnostic tool that assesses the presence of neuropathic pain through self-report and physical examination. Each of the 10-item is answered yes (score 1) or no (score 0). A total score is computed by summing all 10 items and a score ≥ 4 is indicative of the presence of neuropathic pain. The DN4 has good sensitivity (82.9%) and specificity (89.9%), as well as good construct validity and reliability.^22^

Patients were categorized as having neuropathic pain if they had a score ≥ 4 on the DN4^22^ that suggested the presence of neuropathic pain and received a neuropathic pain diagnosis from the treating doctor. Patients were categorized as having nonneuropathic pain if they had a score < 4 on the DN4 and a medical nonneuropathic pain diagnosis. Last, patients were categorized as having a mixed evidence of neuropathic pain when there were conflicting results between the DN4 score and the physician’s pain diagnosis (e.g., DN4 ≥ 4 and a nonneuropathic pain diagnosis).

#### Brief Pain Inventory–10

The BPI-10^23^ is a modified version of the seven-item BPI^24–26^ and contains a total of 10 items assessing pain interference on various aspects of daily living. Participants are asked to rate on a scale from 0 (*does not interfere*) to 10 (*completely interferes*) the extent to which pain has interfered in the past 7 days with general activity, mood, mobility, normal work, relationships with others, sleep, enjoyment of life, self-care, recreational activities, and social activities. Items are summed and averaged to create a mean score, with higher scores indicating greater pain interference. The BPI has been shown to have good validity and sensitivity to change in CP patients receiving MPT.^27^ The BPI has been translated into French using a forward–backward translation method.^28^

#### Chronic Pain Sleep Inventory

The Chronic Pain Sleep Inventory (CPSI)^29^ is a five-item questionnaire that assesses sleep quality in patients with CP. The first four items assess sleep onset, need for sleep medication, wake after sleep, and early morning awakening; participants are asked to rate each item on a scale from 0 (*never*) to 10 (*always*). The last item assesses subjective global sleep quality and participants rate this item on a scale from 0 (*very bad*) to 10 (*excellent*). Three of the sleep items (sleep onset, wake after sleep, and early morning awakening) can be summed to compose a sleep quality index,^29^ with higher scores indicating worse sleep quality. The CPSI has excellent internal consistency (α = 0.90), adequate convergent validity (*r* ≥ 0.50) between CPSI items, discriminant validity (*r* < 0.50 with measures of disability and quality of life), and adequate sensitivity to change.^29^ This questionnaire has been translated into French in the context of the QPR project using a forward–backward translation method by native French and English speakers.

#### Beck Depression Inventory–I

The Beck Depression Inventory-I (BDI-I)^30,31^ is a 21-item scale that assesses depressive symptomology (both psychological and somatic symptoms). For each question, patients are asked to select one of four statements (rated from 0 to 3) that best describe the way they are feeling. Total score is computed by summing all items, with higher scores indicating higher levels of depressive symptomology. The BDI-I has excellent reliability and validity in a wide range of medical populations.^32,33^

#### Pain Catastrophizing Scale

The Pain Catastrophizing Scale (PCS)^34,35^ is a valid and reliable 13-item scale measuring pain-related rumination, magnification, and helplessness. Participants rate each item on a scale from 0 (*not at all*) to 4 (*all the time*), for a total score of 52. Higher total scores indicate greater tendency to catastrophize about pain. Cronbach’s alpha of 0.87 for the total scale is satisfactory. The scale also has good convergent validity with measures of anxiety (*r =* 0.32) and negative affect (*r* = 0.32). Ten-week test–retest reliability showed good reliability (*r* = 0.70).^34,36,37^

#### Short-Form-12 Health Survey Version 2

The Short-Form-12 Health Survey Version 2 (SF-12v2)^38^ is a valid and reliable 12-item scale that assesses quality of life (QOL) in terms of physical health and mental health. For each item, patients are asked to check the box that best describes their condition; answer options vary from one question to the next. The SF-12v2 generates norm-based scores for eight different domains as well as two composite summary scores representing physical health– and mental health–related QOL. Higher scores indicate better QOL. The SF-12v2 has good test–retest reliability (*r* = 0.76–0.89) and internal consistency (α = 0.81–0.84).

### Data analysis

Means and standard deviations along with frequency tables were used to describe participants’ characteristics. Independent Student’s *t* tests and Fisher’s exact tests were employed to compare the baseline characteristics of patients who did and did not complete any follow-up time points. Considering that even small differences can reach statistical significance in large sample sizes,^39,40^ effect sizes of group differences were also calculated using Cohen’s *d* for continuous variables^41^ and the phi (φ) statistic for dichotomous variables.^42^ Only differences that reached a *d* value ≥ ±0.4 or a φ value ≥ ±0.3 were considered meaningful and clinically important.^39,40^

### Pain severity trajectories

Pain severity trajectory analyses were conducted in patients who completed the patient questionnaire at baseline and at least one of the follow-ups—that is, at 6, 12, and/or 24 months. Growth mixture modeling for multivariate latent classes (GMM^43–45^) was used to carry out pain trajectory analyses. This longitudinal data analysis method uses latent class membership to estimate discrete trajectories based on multivariate outcomes. R (Ver. i386 3.1.2^46^) was used to empirically examine several models that differed in terms of number of trajectories and model structure using the latent class mixed models (lcmm) package.^47^ The multivariate dependent variables were pain intensity and pain interference. Scores on the NRS-11 representing average pain intensity on the previous 7 days and scores on the BPI-10 (average score on the 10 interference items), both collected at each time point (baseline, 6 months, 12 months, 24 months), were used to estimate pain severity trajectories. Models varied based on the number of trajectories being tested (number of classes was not predetermined and increased until either the fit indices stopped decreasing or the interpretation of data was no longer plausible) and time effects (the use of a linear constant, quadratic, or asymptotic terms). The Akaike information criterion (AIC) and Bayesian information criterion (BIC^48,49^; lowest values indicate a better fit to the data) were used to determine the best model fit. Model selection was also based on interpretability of trajectories^50^ and favored a parsimonious model.

To address the issue of local maxima, three additional models were run with initial starting values specified for all model parameters. These initial starting values were generated based on variations of the parameter values of the retained model.

### Predictive model of pain severity trajectories

Once the final model was selected and patients were assigned to a specific trajectory (assignation is based on the trajectory to which the patient has the highest probability of belonging), trajectories were merged into two groups—improvers vs. non-improvers—based on the slope of their pain trajectory (neutral or positive vs. negative slope). Logistic regression analysis was used to identify baseline characteristics that differentiate improvers from nonimprovers. The following baseline characteristics were considered in the logistic regression model using a forward conditional procedure: age, sex, type of pain (neuropathic, nonneuropathic, mixed evidence), worst pain intensity (NRS-11), depression (BDI-I), sleep quality (CPSI sleep quality index), pain catastrophizing (PCS), and physical and mental health–related quality of life (SF-12v2).

### Pain trajectories as predictors of 24-month outcomes

A multivariate general linear model (GLM) was conducted in order to examine how belonging to the improvers vs. non-improvers classes of pain severity predicted patient outcomes at 24 months in terms of depression, sleep quality, and physical and mental health–related QOL.

### Sample size estimation

#### Pain trajectories

Given that no data are currently available on means, standard deviations, and rates of change between group slopes and intercepts, sample size was estimated based on relevant simulations for growth curve models.^51^ The estimation was made for measurements at four time points with an estimated moderate slope correlation effect size (*r* = 0.50) and a growth curve reliability > 0.85. With these conditions met, a sample size of 500 patients generates a power level > 0.80.

#### Logistic regression analysis and multivariate general linear model

Using G*Power (Ver. 3.1.2), a sample size of 721 participants is required to achieve power = 80% with odds ratio = 1.3 and Pr(*Y* = 1│*X* = 1) H_0_ = 0.2, and alpha = 0.05. A sample size of 244 participants is required to achieve power = 80% with effect size *f*^2^ = 0.05, alpha = 0.05, with two groups and four response variables.

## Results

### Sample characteristics

**Figure 1.**
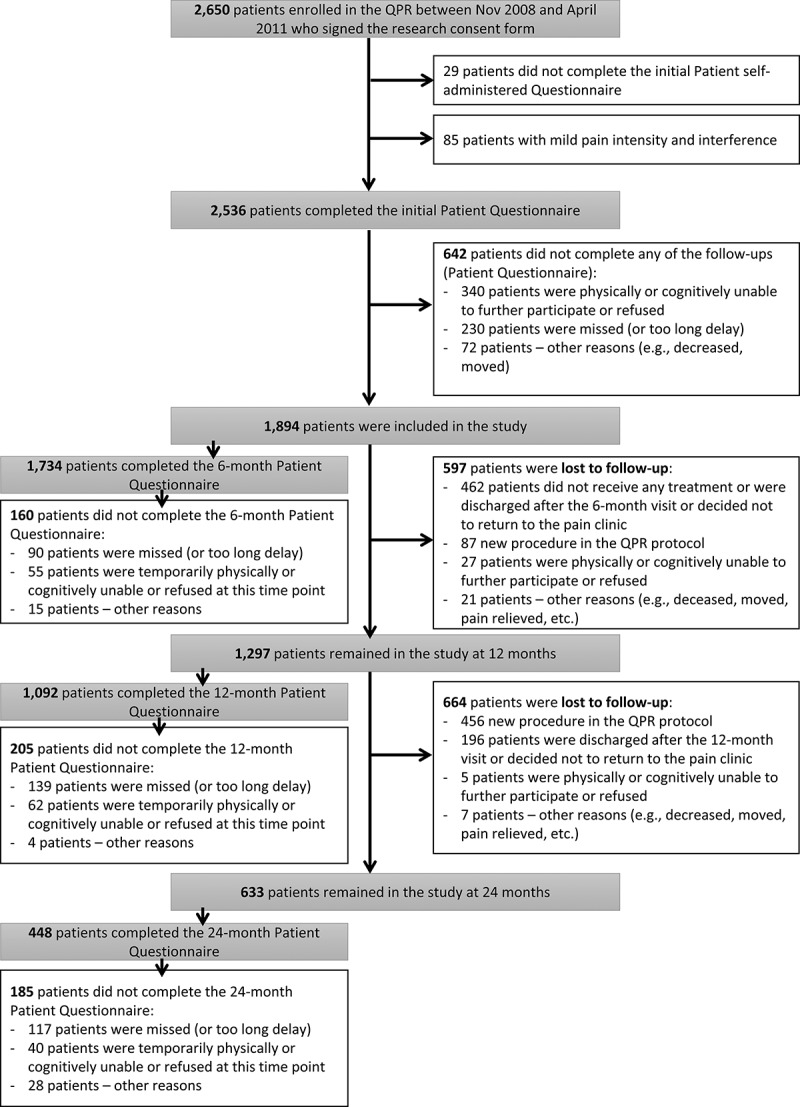
Study flow diagram.

A final sample of 1894 participants was retained for this study (Figure 1); these participants reported initial pain intensity and interference in the moderate-to-severe range (NRS and/or BPI mean score ≥ 4). Comparisons between patients who completed enough time points to be included in pain trajectory analyses (≥2; *n* = 1894) and those who did not (*n* = 756) revealed no clinically significant difference in age, sex, pain duration, baseline measures of average or worst pain intensity, pain interference, sleep quality, depression, and physical and mental health–related QOL (all *P* > 0.05 and/or Cohen’s *d* value < 0.4/φ < 0.3).

**Figure 2.**
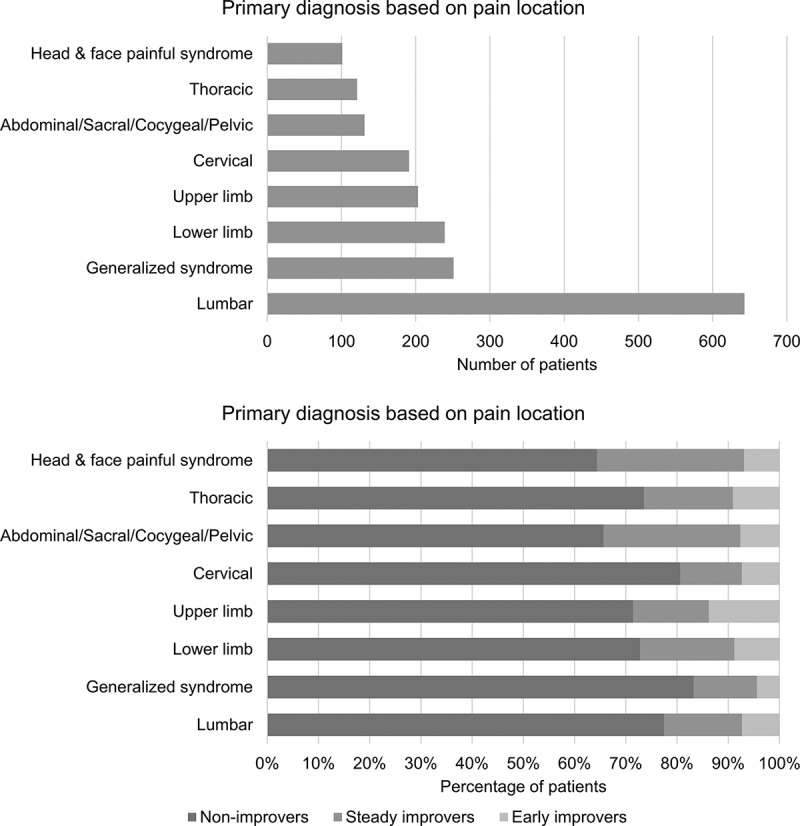
Pain diagnoses in the overall sample and in each pain trajectory.

Mean age of the participants (*n* = 1894) was 53.18 years (SD = 14.0) and 60.3% were female. Average pain duration was 6.72 years (SD = 8.4). Figure 2 shows the patients’ primary diagnoses made by the physicians at the clinic according to pain location. Approximately one third of patients suffered from chronic lumbar pain. Based on the data collected with the DN4 and the type of pain disorder or its suspected etiology, the estimated percentages of patients who had neuropathic pain, nonneuropathic pain, and mixed evidence of neuropathic pain were 29.4%, 24.0%, and 46.6%, respectively. Other baseline data on patients’ pain characteristics, psychological measures, and QOL summary scales of the SF-12v2 are presented in Table 1.

**Table 1.** Patients’ demographic, pain, and psychological characteristics for the overall sample and in each pain severity trajectory at baseline.^a^

		Pain severity		
	Total (*n* = 1894)	Trajectory 1—non-improvers (*n* = 1430)	Trajectory 2—early improvers (*n* = 313)	Trajectory 3—steady improvers (*n* = 151)
Age, mean (SD)	53.18 (14.0)	53.50 (14.0)	51.57 (13.7)	53.39 (14.1)
Sex, *n* (%)				
Female	1142 (60.3)	858 (60.0)	187 (59.7)	97 (64.2)
Male	752 (39.7)	572 (40.0)	126 (40.3)	54 (35.8)
Type of pain, *n* (%)				
Neuropathic pain	486 (29.4)	362 (28.0)	77 (31.8)	47 (39.2)
Nonneuropathic pain	396 (24.0)	299 (23.2)	67 (27.7)	30 (25.0)
Mixed evidence	771 (46.6)	630 (48.8)	98 (40.5)	43 (35.8)
Mean (SD)				
Pain duration (years)	6.72 (8.4)	6.98 (8.4)	6.88 (9.3)	3.96 (5.6)
Average pain	6.95 (1.7)	7.37 (1.5)	4.95 (1.4)	7.12 (1.7)
Worst pain	8.39 (1.5)	8.66 (1.3)	7.11 (1.6)	8.38 (1.4)
BPI-10	5.98 (2.0)	6.40 (1.9)	4.24 (1.8)	5.63 (1.9)
CPSI index	18.05 (8.5)	19.40 (8.0)	12.76 (8.4)	16.19 (8.6)
BDI-I	19.38 (10.3)	20.76 (10.4)	14.94 (8.5)	15.62 (9.6)
PCS	30.49 (12.4)	32.41 (12.1)	23.39 (10.5)	27.09 (13.1)
QOL physical health	28.28 (8.5)	27.10 (7.8)	32.66 (9.7)	30.31 (8.9)
QOL mental health	40.00 (11.6)	38.80 (11.4)	44.10 (11.2)	42.76 (11.9)

^a^Average pain: average pain intensity score over the past 7 days measured on the NRS-11; Worst pain: average worst pain intensity score over the past 7 days measured on the NRS-11; BPI-10: average score on the interference scales of the Brief Pain Inventory–10; CPSI index: Sleep quality index of the Chronic Pain Sleep Inventory; BDI-I: total score on the Beck Depression Inventory–I; PCS: total score on the Pain Catastrophizing Scale; QOL physical health: norm-based physical health summary score of the SF-12v2; QOL mental health: norm-based mental health summary score of the SF-12v2. BPI-10 = Brief Pain Inventory–10; CPSI = Chronic Pain Sleep Inventory; BDI-I = Beck Depression Inventory–I; PCS = Pain Catastrophizing Scale; QOL = quality of life; SF-12v2 = Short-Form-12 Health Survey Version 2.

When patients were asked to rate the intensity of their average pain in the past 7 days, the overall mean NRS score at baseline was at the upper end of the range for moderate pain (mean = 6.95, SD = 1.7). Their scores then slightly decreased at the 6-month follow-up (mean = 6.21, SD = 2.2) and remained stable on the average at the 12- and 24-month follow-ups. The same was true for the interference scores (baseline: mean = 5.98, SD = 2.0; 6 months: mean = 5.29, SD = 2.4).

### Pain trajectories

Eighteen different GMM models were evaluated; they varied from one another based on the number of classes being tested (between one and six classes were tested) and on the use of a linear constant, mixed linear and quadratic, or mixed linear and asymptotic terms. Table 2 provides details and fit indices for all models tested. The final model retained, based on fitness indicators and theoretical soundness, was a three-trajectory model with linear and quadratic terms (AIC = 40 212.2; BIC = 40 334.3). Even though the four- and five-trajectory models with linear and quadratic terms had slightly better model fit indicators and the three-trajectory model with the linear and asymptotic terms had equal model fit indicators, these models contained trajectories with classes representing less than 5% of the sample. Research showed that indicators perform poorly when models contain such small classes.^52^ A three-trajectory model with linear and quadratic terms was thus retained to improve fit and interpretability while offering a more parsimonious solution. The additional three models that were run to control for local maxima returned parameters values identical to the final model parameter estimates to the first or second decimal value (except for the standard error of the autoregressive process, which had a 0.4 difference between the highest and lowest values across the four models). As such, this confirmed that the final solution was not due to local maxima.

**Table 2.** Fit indices for all growth mixture models tested.^a^

	Linear only		Linear and quadratic		Linear and asymptotic	
Number of trajectories	AIC	BIC	AIC	BIC	AIC	BIC
1	40 669.9	40 736.5	40 584.7	40 662.4	40 456.1	40 533.7
2	40 475.0	40 558.2	40 264.1	40 363.9	40 237.6	40 337.5
3	40 399.2	40 499.1	40 212.2^a^	40 334.3^a^	40 212.8	40 334.8
4	40 394.1	40 510.6	40 220.2	40 364.4	40 188.0	40 332.2
5	40 389.7	40 522.8	40 190.1	40 356.5	40 181.7	40 348.1
6	40 410.2	40 560.0	40 236.5	40 425.0	40 185.3	40 373.9

^a^Linear and quadratic with three classes was the best model fit (optimal model when both AIC and BIC improve compared to the previous model and when all classes *n* > 5%). AIC = Akaike information criterion; BIC = Bayesian information criterion.

**Figure 3.**
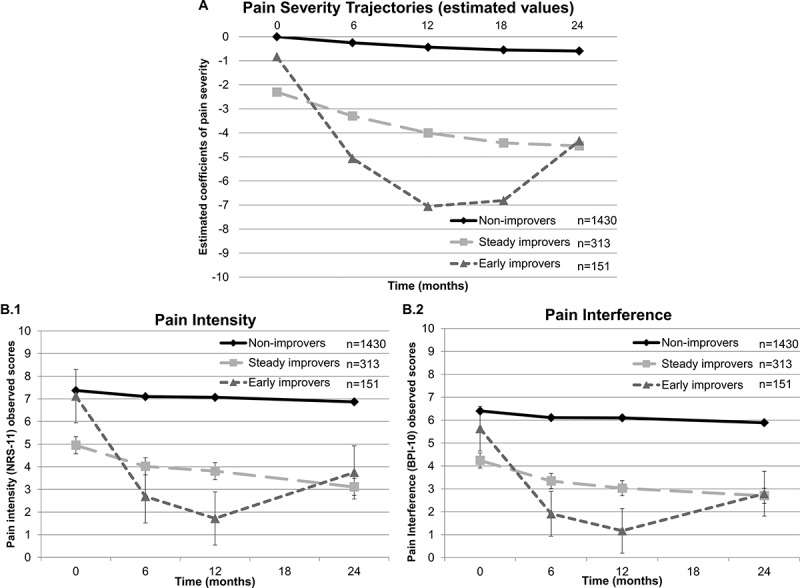
Growth mixture modeling analyses. Results of the GMM analyses showing three different pain severity trajectories. Panel A represents the trajectory model with parameter estimates. The *Y*-axis represents a linear combination of the outcome variables (NRS-11 and BPI-10) similar to a factor score or a weighted average. By default, the intercept of the null model is set at 0. Trajectory 1 (non-improvers) represents patients with initially elevated pain severity who do not improve over time. Trajectory 2 (steady improvers) represent patients with initially moderate pain severity who demonstrate a constant improvement in their pain severity over time. Last, trajectory 3 (early improvers) represent patients with initially elevated pain severity who rapidly improve over the first 6 months and then experience a loss in their improvement so that their pain severity returns to a moderate level. These patients, however, continue to report significantly lower levels of pain severity at 24 months compared to baseline. Panel B.1 shows the pain intensity observed values across time for each trajectory and panel B.2 shows the pain interference observed values across time for each trajectory. It is important to note that the classification of patients in a trajectory is based on a probabilistic model and is provided here as a way to illustrate differences between pain trajectories.

Figure 3 illustrates all three pain severity trajectories across time, with two trajectories (nos. 2 and 3) representing patients improving with treatment and one trajectory (no. 1) representing patients not improving with treatment. Characteristics and parameter estimates of the regression equations for each trajectory are shown in Table 3. Pearson’s chi-square test showed a statistically, χ^2^ (df = 4) = 18.84, *P* = 0.001, but not clinically significant (Cramér’s *V* = 0.07) difference in trajectory membership and clinic site.

**Table 3.** Characteristics and parameter estimates of the regression equations for each pain severity trajectory.

			Slopes		Predicted values			
Trajectory	*n*	Intercept	Linear	Quadratic	Baseline	6 Months	12 Months	24 Months
1 (Non-improvers)	1430	0^a^	−0.05**	0.001	0	−0.25	−0.44	−0.59
2 (Steady improvers)	313	−2.30**	−0.19**	0.004*	−2.30	−3.30	−4.00	−4.54
3 (Early improvers)	151	−0.83*	−0.89**	0.031**	−0.83	−5.06	−7.06	−4.33

^a^Intercept for first trajectory set at 0. *P < 0.05. **P < 0.01.

As shown in Figure 1, only a small proportion of patients did not complete the 12- and/or 24-month patient questionnaire because their pain was relieved (*n* = 17) and thus they were discharged from the MPT clinic. These patients were more likely to be classified in trajectories 2 (*n* = 5) and 3 (*n* = 9; improvers) and less likely to be represented in trajectory 1 (*n* = 3). Results of Pearson’s chi-square test showed no clinically significant differences in trajectory membership across the three MTP clinics (*P* = 0.001; φ < 0.3).

Figure 2 provides for each trajectory details of patients’ pain diagnoses based on its location. Because the number of patients having a particular diagnosis in some of the trajectories was very small (*n* < 5), it was not possible to assess statistical significance of pain diagnosis differences across trajectories. There were no significant sex (χ^2^ = 1.07, *P* = 0.585) or age, F(2, 1891) = 2.49, *P* = 0.083, differences across pain trajectories.

### Predictive model of pain severity trajectories

Results of the logistic regression analysis (χ^2^ = 264.00, *P* < 0.001, percentage of correct prediction = 80.1%) showed that seven baseline characteristics were significant predictors of pain severity trajectory, namely, age, type of pain (neuropathic, nonneuropathic, mixed evidence), worst pain intensity, depression, physical health–related quality of life, sleep, and pain catastrophizing (see Table 4). The model had a sensitivity of 23.60%, a specificity of 96.17%, positive predictive value of 63.64%, and negative predictive value of 81.60% in identifying treatment improvers (see Table 5).

**Table 4.** Logistic regression analysis with baseline characteristics as predictors of pain severity classes (improvers vs. non-improvers).^a^

Variables^a^	B	SE	Wald	df	*P* value	Exp(B)	95% CI^c^	
Constant^b^	3.60	0.61	34.75	1	<0.001	36.59		
Age	−0.01	0.01	7.80	1	0.005	0.99	0.98	−0.996
Type of pain			8.52	2	0.014			
NP(1)	−0.32	0.18	3.22	1	0.073	0.73	0.51	−1.03
NP(2)	−0.45	0.16	8.36	1	0.004	0.64	0.47	−0.87
Worst NRS	−0.36	0.05	53.61	1	<0.001	0.70	0.63	−0.77
BDI-I	−0.03	0.01	9.78	1	0.002	0.97	0.96	−0.99
CPSI	−0.03	0.01	13.28	1	<0.001	0.97	0.95	−0.99
PCS	−0.02	0.01	9.94	1	0.002	0.98	0.97	−0.99
QOL physical health	0.02	0.01	8.52	1	0.004	1.02	1.01	−1.04

^a^Type of pain: neuropathic pain is the reference category; worst NRS: worst pain intensity on the numeric rating scale; QOL physical health: norm-based physical summary scale of the SF-12v2. Variables at baseline considered in the model: age, sex, type of pain (NP, non-NP, mixed evidence of NP), worst pain intensity in the past 7 days, depression, sleep quality, pain catastrophizing, and physical and mental health–related quality of life.

^b^Omnibus test of model coefficients: χ^2^ = 264.00, *P* < 0.001. NP = neuropathic pain; NP(1) = non-NP; NP(2) = mixed evidence of NP; NRS = Numerical Rating Scale; BDI-I = Beck Depression Inventory–1; CPSI = Chronic Pain Sleep Index–Sleep Quality; PCS = Pain Catastrophizing Scale; QOL = quality of life; SF-12v2 = Short-Form-12 Health Survey Version 2.

^c^CI = confidence interval

**Table 5.** Classification of patients based on the results of the logistic regression analysis.

		Predicted values		
Observed values		Non-improvers	Improvers	% Correct
	Non-improvers	1206	48	96.2
	Improvers	272	84	23.6
	Overall %			80.1

### Pain trajectory membership as predictor of 24-month outcomes

**Figure 4.**
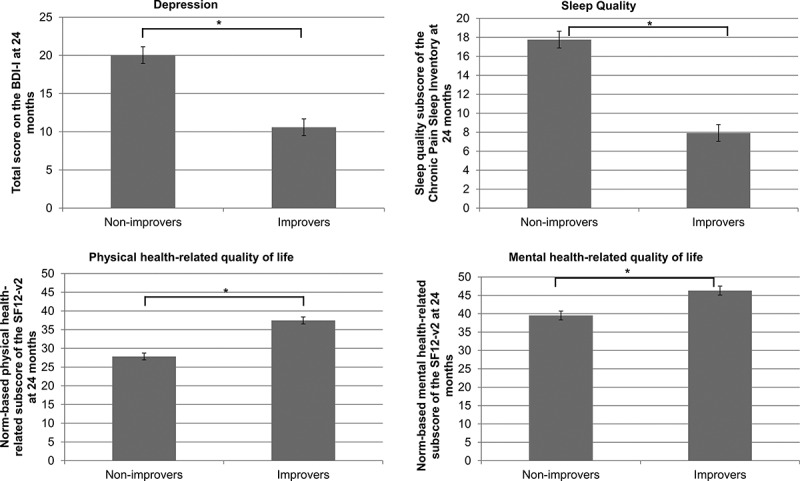
Multivariate GLM. Results of the multivariate GLM examining differences in depression (BDI-I total score), sleep disturbances (total score on the CPSI sleep quality index), and QOL (norm-based score on the physical and mental health–related QOL summary score of the SF12-v2) at 24 months across pain severity trajectories.

Results of the multivariate GLM showed an overall significant model, Pillai’s trace = 0.305; F(4, 442) = 48.54, *P* < 0.001. Compared to non-improvers, improvers reported significantly fewer symptoms of depression, as well as better sleep quality and physical and mental health–related QOL (all *P* values < 0.001) at 24 months. Figure 4 further illustrates these associations.

## Discussion

Results of this study, which used real-world data and latent trajectory analysis, suggest that responses to tertiary care multidisciplinary pain treatment clearly vary considerably in patients suffering from chronic non-cancer pain. One quarter of patients showed a significant improvement in their pain severity levels, a composite of pain intensity and interference. Compared to patients in the non-improving trajectory, patients in the improving trajectories were younger, were more likely to suffer from neuropathic pain, had lower worst pain intensity, had lower sleep disturbances and depression scores at baseline, had a lower tendency to catastrophize, and had better physical health–related QOL at baseline. Patients were correctly classified as improvers or non-improvers 80% of the time. Improvers also reported better pain-related outcomes at 24 months compared to non-improvers in terms of other dimensions of the pain experience (sleep, depression, and physical and mental QOL).

### Pain severity trajectories

Overall, patients experienced on average a small decrease in pain intensity and interference within the first 6 months of attending an MPT clinic. The reported pain intensity and interference remained relatively the same over the following 18 months. However, results suggest that looking only at the overall sample trajectory leads to an inaccurate interpretation of treatment effectiveness, whereas looking at underlying latent subgroup trajectories allows for a more comprehensive view of how pain evolves over time. Exploration of inter- and intra-individual variability in pain severity trajectories revealed the presence of three distinct groups of patients that differed in terms of initial level of pain severity and rates of improvement over time. This pain trajectory model showed that only one quarter of patients (24.5 %) experienced a significant decline in pain severity over 24 months. One third of these patients who reported an improvement in their pain severity did not successfully maintain their gains over time. In fact, this specific subgroup (early improvers) significantly benefited from MPT over the first year, but their pain severity started to gradually worsen beyond that time. It is possible that for a subgroup of patients, either the interventions lost their effect (e.g., opioid tolerance) or they did not persist in using the learned skills (e.g., physiotherapy exercises, relaxation techniques, pain management tools). The most important subgroup in terms of proportion of patients reported constantly high levels of pain severity during the same period. Three quarters of patients reported an elevated pain severity that remained stable over time. The obtained results with the pain trajectory analysis point to the importance of considering patient heterogeneity and intra-individual differences when examining treatment efficacy and effectiveness. From a methodological point of view, these results are consistent with other studies suggesting that averaging pain scores across groups or samples results in the loss of accuracy and within-individual variations.^13,14^ This is even more important in the context where the health care system is moving toward a patient-oriented approach as recommended by the Institute of Medicine^53^; treatment guidelines, and recommendations should more appropriately be based on patient-centered empirical evidence.

### Predictive model of pain severity trajectories

Compared to patients in the non-improving trajectory, patients in the improving trajectories were younger and tended to suffer from neuropathic pain; they also had lower worst pain intensity at baseline, fewer sleep disturbances and lower depression scores, lower tendency to catastrophize, and better physical health–related QOL. These results are important in that they suggest that in addition to pain severity, these characteristics measured at baseline can help predict which pain trajectory patients are most likely to belong to (improving vs. non-improving trajectories). The model suggests that as early as the first visit, specific criteria can be used to identify patients who might not respond to conventional MPT approaches. It is also possible to use these criteria to identify patients who are more likely to benefit from an MPT and prioritize these patients in terms of access to care. Additional research aimed at identifying other factors that would improve the predictive value of the model is required before such approaches are implemented. These results also open the door to the elaboration and evaluation of alternative treatments that might be more cost-effective for this subgroup. Early identification of these patients offers the opportunity to adjust treatment plan and design interventions that best fit their clinical presentation.

#### Age

Age is an important factor in determining treatment responses such that younger age offers a more optimistic prospect in terms of treatment response. It is possible that younger adults are offered different treatment approaches compared to older ones because of the specific characteristics of the elderly population (e.g., differences in drug distribution in the body^54^ leading to different analgesic prescription regimens).

#### Worst pain intensity

Compared to non-improvers, improvers reported lower worst pain intensity. These results suggest that perhaps patients who experience lesser peaks in pain intensity (worst pain scores) might show better treatment responsiveness. It has also been shown that pain recall is influenced by peak pain intensity and recent pain intensity.^55,56^ As such, it is possible that the presence of higher pain intensity scores influences patients’ pain experiences, leading to higher pain severity reports.

#### Pain catastrophizing, depression, sleep disturbances, and physical health–related QOL

Pain catastrophizing has been associated with both experimental and clinical pain experiences,^57^ and it has been shown that reduction in levels of pain catastrophizing is associated with better treatment outcomes.^58^ Similarly, a negative relation has been found between the presence of depression and decreased pain treatment responses,^59^ whereas sleep disturbances are associated with hyperalgesia through descending modulatory systems.^60^ The presence of pain catastrophizing and depression is often associated with increased disability and physical functioning,^57^ thus resulting in lower physical health–related QOL. As such, results from this study are consistent with the existing literature, but they go further in showing that despite similar pain severity at baseline between patients in trajectories 1 and 3, the presence of depression, pain catastrophizing, sleep disturbances, and QOL predicted poor treatment response. In other words, these results suggest that factors other than pain severity predict poor treatment responses. Knowing that patient condition deteriorates while waiting for treatment,^61^ it would be interesting for future research to examine whether these psychological characteristics should be taken into account at triage to determine whether earlier interventions in high pain severity and psychologically distressed patients would allow them to move to an improving pain severity trajectory.

#### Sex and mental health–related QOL

These two factors were not significantly associated with treatment response (improvers vs. non-improvers). The absence of sex differences is consistent with a study of CP patients on waitlist for MPT that found that women and men do not differ in terms of their pain experience (pain intensity, functioning, QOL, and well-being).^62^ Results in the literature are also mixed regarding the presence of sex differences in response to pharmacologic and nonpharmacologic pain treatments.^63^ Though in this study males and females were not differently assigned to trajectories, it would be interesting to compare treatment assignation; that is, whether males and females are being prescribed different treatments for the same symptom presentation. Depression, but not mental health–related QOL, was associated with treatment response. It is possible that specific features of depression (either depressive symptoms; e.g., sadness, apathy, fatigue, worthlessness) or accompanying features (e.g., cognitive impairment) are directly contributing to the pain experience through common maintaining factors or are indirectly influencing treatment response by decreasing one’s ability to fully comply with treatment. It would be interesting in future research to examine what mechanisms associated with depressive episodes but not part of overall mental health QOL are associated with treatment response.

### Twenty-four-month outcomes

Being an improver or non-improver was significantly associated with patient outcomes at 24 months. More specifically, results showed that improvers had lower levels of depressive symptomology, as well as better sleep quality and QOL (both physical and mental health–related) at follow-up. These results are consistent with those observed at baseline, suggesting that patients’ pain and psychological characteristics play an important role in treatment responsiveness. This raises the possibility that these factors are influencing each other and covary across time. It would be interesting to examine this issue in future studies.

## Study strengths and limitations

This study uses a real-world data set of nearly 2000 patients attending one of the dedicated centers of expertise in the multidisciplinary treatment of CP in Quebec. The results thus contribute to our knowledge of the effectiveness of multidisciplinary pain management by stressing the importance of the heterogeneity of treatment responses. Nonetheless, there are some limitations to this study. First, drop-out rates at 12 and 24 months were high; however, the majority of the drop-outs were systematic (e.g., changes in procedures so that 24-month follow-ups were no longer carried out starting in April 2012). It is possible, however, that this might have influenced the results. Second, pain scores were obtained at baseline as well as 6, 12, and 24 months after initiating treatment. Though these scores sufficiently informed the pain trajectory model, they did not capture the pain severity changes that might have occurred acutely between the time points (within the first 6 months and at later times). It would be interesting for future studies to collect real-time data at closer time points during treatment. Third, it was not possible to examine distribution of precise pain diagnoses across pain trajectories given the too-small sample size of some diagnostic categories. Fourth, the statistics presented here represent the characteristics of patients who are long-term users of MPT, which might limit the generalizability of the results to all patients attending an MPT clinic (e.g., patients who have one or two consultations at an MPT clinic are then returned to their general practitioner for ongoing management). Finally, it was not possible within this study to control for variations in MPT approaches across participating sites. However, our results revealed no significant differences in pain trajectory membership across participating centers, suggesting that the observed latent trajectories are not an artefact of treatment centers.

Despite its limitations, this study is the first to our knowledge to examine pain severity trajectories in a large heterogeneous sample of patients with CP attending an MPT clinic. The results revealed the presence of subgroups of patients that differed in terms of their clinical evolution over a 2-year period as well as in terms of their baseline and 24-month pain and psychological characteristics (e.g., pain duration, depression levels). These results are important in that they identified several subgroups of patients who did not improve over the course of treatment. Early identification of these patients, through examination of characteristics such as worst pain intensity, sleep disturbances, depression, pain catastrophizing, and QOL, can provide valuable information about prognosis. Future research directions include the examination of different treatment approaches that could best fit the patients’ pain trajectories.
